# Craniofacial ontogeny in *Centrosaurus apertus*

**DOI:** 10.7717/peerj.252

**Published:** 2014-02-13

**Authors:** Joseph A. Frederickson, Allison R. Tumarkin-Deratzian

**Affiliations:** Temple University, Department of Earth and Environmental Science, Philadelphia, PA, USA

**Keywords:** Dinosauria, Ceratopsia, Ontogeny, Cladistics, Centrosaurus, Growth, Fossil

## Abstract

*Centrosaurus apertus*, a large bodied ceratopsid from the Late Cretaceous of North America, is one of the most common fossils recovered from the Belly River Group. This fossil record shows a wide diversity in morphology and size, with specimens ranging from putative juveniles to fully-grown individuals. The goal of this study was to reconstruct the ontogenetic changes that occur in the craniofacial skeleton of *C. apertus* through a quantitative cladistic analysis. Forty-seven cranial specimens were independently coded in separate data matrices for 80 hypothetical multistate growth characters and 130 hypothetical binary growth characters. Both analyses yielded the max-limit of 100,000 most parsimonious saved trees and the strict consensus collapsed into large polytomies. In order to reduce conflict resulting from missing data, fragmentary individuals were removed and the analyses were rerun. Among both the complete and the reduced data sets the multistate analyses recovered a shorter tree with a higher consistency index (CI) than the additive binary data sets. The arrangement within the trees shows a progression of specimens with a recurved nasal horn in the least mature individuals, followed by specimens with straight nasal horns in relatively more mature individuals, and finally specimens with procurved nasal horns in the most mature individuals. The most mature individuals are further characterized by the reduction of the cranial horn ornamentations in late growth stages, a trait that similarly occurs in the growth of other dinosaurs. Bone textural changes were found to be sufficient proxies for relative maturity in individuals that have not reached adult size. Additionally, frill length is congruent with relative maturity status and makes an acceptable proxy for ontogenetic status, especially in smaller individuals. In adult-sized individuals, the fusion of the epiparietals and episquamosals and the orientation of the nasal horn are the best indicators of relative maturity. This study recovers no clear evidence for sexually specific display structures or size dimorphism in *C. apertus*.

## Introduction

*Centrosaurus apertus* (Lambe, 1904) is one of the best represented Late Cretaceous dinosaurs, with a fossil record that includes dozens of partial to complete skulls, articulated skeletons, and hundreds of isolated cranial and postcranial bones. This rich fossil record can partly be attributed to the presence of multiple monodomnant bonebed assemblages, which contain specimens that range from small juveniles to full-grown adults (Ryan & Evans, 2005). The largest of these individuals demonstrate variability in the size and shape of horns found on the snout, supraorbital, and frill regions. This morphological variability within *C. apertus* populations has been attributed to multiple factors, including ontogeny (Sampson, Ryan & Tanke, 1997; Ryan *et al.*, 2001), sexual dimorphism (Dodson, 1990), and individual variability (Ryan *et al.*, 2001). Of these hypotheses, ontogeny has been the main focus of recent studies into this morphological diversity (Sampson, Ryan & Tanke, 1997; Ryan *et al.*, 2001; Brown, Russell & Ryan, 2009; Tumarkin-Deratzian, 2010). *C. apertus* makes an ideal subject for an ontogenetic study, because of its relatively complete fossil record and stratigraphic isolation from most if not all other centrosaurines (Ryan & Evans, 2005; Mallon *et al.*, 2012).

Sampson, Ryan & Tanke (1997) and Ryan *et al.* (2001) reconstructed growth stages in *Centrosaurus apertus* based upon disarticulated bonebed material. Both studies demonstrated that as individuals matured, the skull changed dramatically in appearance. These analyses used size, as well as a combination of qualitative observations of cranial ornamentation development and bone textural changes, to determine relative maturity. These proxies can all be used independently to test relative maturity (Sampson, Ryan & Tanke, 1997; Ryan *et al.*, 2001). However, each of these relative aging techniques has limitations. Size, for example, may be affected by environmental stresses (e.g., Leafloor, Ankney & Rusch, 1998) and becomes useless for relative aging once an animal has reached its largest skeletal proportions. Cranial ornamentations have also classically been used to determine the ontogenetic status of centrosaurines; however, many of these structures do not develop fully until near-maximum sizes are achieved (Sampson, Ryan & Tanke, 1997), and often these data conflict with relative size observations (e.g., larger skulls may have poorly developed cranial ornamentations in contrast to smaller ones (Dodson, 1990)). Suture closure can be an important ontogenetic indicator in vertebrates (e.g., Brochu, 1996), with increasing frequency of fusion generally indicating increasing age (Sampson, Ryan & Tanke, 1997). However, some of the bones in the skull of *C. apertus* remain unfused, even in the largest known individuals (Ryan *et al.*, 2001). Further, suture closure has been hypothesized to be an unreliable ontogenetic indicator in the related genus *Triceratops* (Scannella & Horner, 2011). Bone texture changes offer a size-independent test of relative maturity; however, with only three major cranial stages identified, it is only possible to create very broad ontogenetic groups using this technique. Furthermore, studies on extant archosaurs indicate that the rate and timing of textural changes may vary in different bones of the same individual (Tumarkin-Deratzian, Vann & Dodson, 2006).

Previous ontogenetic studies of *Centrosaurus apertus* have been hindered by the inconsistencies between growth proxies, which have only allowed for the identification of imprecise anatomical changes, especially among full-sized individuals (Ryan *et al.*, 2001). The problematic and often conflicting inferences of maturity from these data mean none of these techniques alone can reliably and unequivocally reconstruct a complete growth series for *C. apertus*. However, if each proxy for growth is integrated equally into a single hypothesis on the development of *C. apertus*, then a growth series could be determined with increased resolution. A method to combine independent ontogenetic proxies into a single analysis was published by Brochu (1996, and references therein) utilizing a quantitative cladistic methodology. Brochu’s (1996) technique evaluates the consistency of separate lines of ontogenetic evidence (e.g., size, the development of cranial ornamentations, bone texture) with equal weight, and algorithmically derives a testable hypothesis for the sequential accumulation of growth characters. Tumarkin-Deratzian, Vann & Dodson (2006), Tumarkin-Deratzian, Vann & Dodson (2007) used this technique as a proxy for age in the American alligator (*Alligator mississippiensis*) and the Canada goose (*Branta canadensis*), although it should be noted that only the Canada goose analysis yielded a useful growth series of consistent ontogenetic character changes. Recently the method has also been applied to non-avian dinosaurs, with hypothetical growth series proposed for *Tyrannosaurus rex* (Carr & Williamson, 2004), *Albertosaurus sarcophagus* (Carr, 2010), and *Triceratops* and *Torosaurus* (Longrich & Field, 2012).

The primary objective of this study is to combine multiple different lines of ontogenetic proxies for growth in *Centrosaurus apertus* into a cladistic analysis. If a hierarchical signal exists within the known fossil record, then a growth series for this taxon can be reconstructed. Once a growth sequence of ontogenetically informative characters is recovered, characters that are individually variable within the population can be identified. Further, the presence of sexual dimorphism can be tested once the ontogenetic progression is known. Sexual dimorphism, if present, should appear as variable growth characters that are only found in roughly half of the most mature (adult) specimens. Without first determining the ontogenetic progression it is difficult, if not impossible, to distinguish growth characters from other forms of variation.

## Methodology

This study follows and modifies the method used by Brochu (1996). Specimens with different morphologies were identified in the primary literature and through first-hand observations in order to define potential hypothetical growth characters. The study sample was restricted to *Centrosaurus* specimens derived from the lower 30 m of the Dinosaur Park Formation, representing less than a one million year range (Eberth, 2005). This stratigraphic constraint reduced potential phylogenetic effects by limiting the analysis to specimens found only in similar depositional environments, which produces the best approximation of a single species of *Centrosaurus*. This stratigraphic restriction is ideal because it contains limited geologic overlap with other centrosaurine species (Ryan & Russell, 2005). It should be noted, however, that this restriction represents the vast majority of the known geologic range of *C. apertus*.

**Figure 1:**
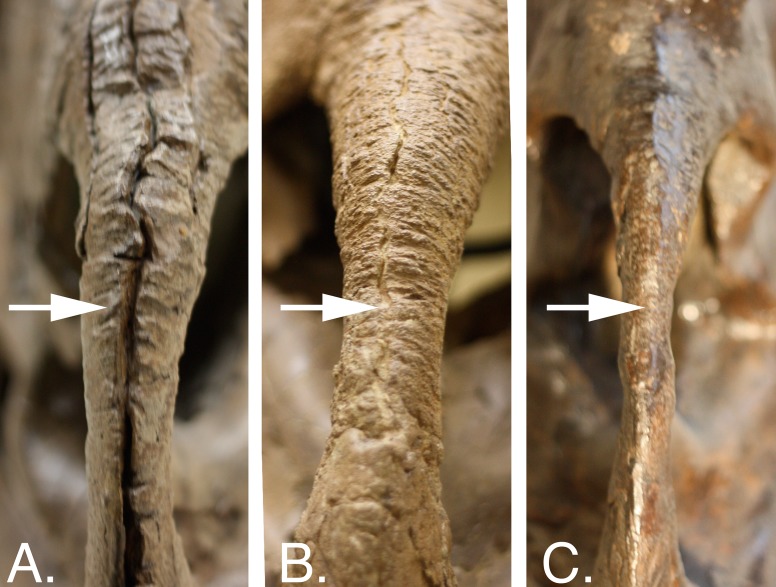
Bone fusion character states for the middle premaxillary suture (white arrow) in rostral view. (A) CMN 348, open; (B) UALVP 11735, partially obliterated.; (C) YPM 2015, fully obliterated. Images not to scale. Division of Vertebrate Paleontology, YPM 2015. Courtesy of the Peabody Museum of Natural History, Yale University, New Haven, Connecticut, USA.

A majority of the growth characters used in this study fall into three main categories: bone fusion, bone texture, and development of ornamentations. These character types have been used as proxies for growth by other authors (Dodson, 1990; Sampson, Ryan & Tanke, 1997; Ryan *et al.*, 2001; Brown, Russell & Ryan, 2009; Tumarkin-Deratzian, 2010). Bone fusion refers to the closure of sutures seen externally between adjacent bones. Scoring for these characters follows Ryan *et al.* (2001): sutures were considered open if bones were separate along their entire margin of contact, partially obliterated if the bones were fused incompletely or if a deep groove or trough was still present along the margin, and fully obliterated if the two bones were conjoined into a single unit with a surface nearly level with the surrounding bone (Fig. 1). Bone textural changes refer to the sequence of three distinct surficial bone textures (striated/long-grained, mottled, or adult/rugose) identified as an ontogenetic progression by previous authors (Sampson, Ryan & Tanke, 1997; Ryan *et al.*, 2001; Brown, Russell & Ryan, 2009) and confirmed by histological study (Tumarkin-Deratzian, 2010). Textures were coded based on a modified method of Brown, Russell & Ryan (2009), where the most mature texture that exceeded a 1 × 1 cm^2^ continuous area on the exterior surface of the bone was considered the representative state. Ornamentation refers to the horns, hooks, and processes found on the snout, the jugal, the supraorbital region, and the frill. Ornamentation characters include measurements of structure length, and aspects of structure shape, orientation, and surface texture. The numbering of these processes on the frill follows Sampson, Ryan & Tanke (1997). Other growth characters that are not categorized into the previous three groups include additional structures, such as the presence of foramina and fenestrae which have not previously been used to determine the relative maturity of individual *C. apertus*, but are otherwise seen to be variable within *C. apertus* specimens.

Taphonomic distortion can further contribute to morphological variation in fossils. Many large *Centrosaurus* skulls are asymmetrically distorted (e.g., CMN 348) and/or crushed (e.g., CMN 11837), giving these specimens vastly different shaped skulls, a problem that may not be as readily identifiable in specimens that are modified to a lesser extent. To reduce taphonomic bias in the data set, characters based on overall skull shape and orientation were not used. Characters regarding horn shape and orientation were retained, but not scored in specimens that are obviously distorted.

All specimens and characters were compiled into a data matrix using MacClade v. 3.0 (Maddison & Maddison, 1992), where the nascent (primitive) state for each character is scored 0 and the mature (derived) state is scored 1 or higher. The nascent state was determined using multiple criteria. Specimens with dissimilar character states were first evaluated for major size differences. Size and maturity level are not necessarily equivalent, but specimens that approach average adult sizes are likely to be morphologically more mature than much smaller specimens. In this analysis, size classes follow Ryan *et al.* (2001), with YPM 2015 representing a putative full-grown individual. Skulls less than one-half the maximum length of YPM 2015, or individual bones therein for isolated material, were assumed to possess the nascent condition and were coded 0. Because the length of the skull was used to determine the nascent condition, no other characters related to absolute skull size were included in the analysis. However, characters for display structure (horn) sizes were kept, because these were not included as a measurement of absolute size.

If more than one morphology was present among specimens that were less than one-half the length of YPM 2015, other putative juvenile centrosaurines were used as an analog for the morphology found in *C. apertus* juveniles. The nascent character states for *C. apertus* are assumed to be similar to the character states found in other juvenile centrosaurines, because closely related centrosaurine species are hypothesized to have followed the same growth pattern (Sampson, Ryan & Tanke, 1997). If this second criterion failed to yield a single unequivocal nascent state, the phylogenetic outgroup was used under the assumption that in closely related species ontogeny recapitulates phylogeny, following the methodology of Carr & Williamson (2004) and Carr (2010). For this criterion the morphological feature that most closely resembles the state found in basal neoceratopsians was considered the nascent condition and coded 0.

To address instances of characters with more than one mature state two separate data matrices were constructed based on the different coding methods previously used in these types of analyses, one consisting of multistate characters (e.g., Carr & Williamson, 2004) and the other including only additive binary characters (e.g., Carr, 2010), in order to determine which coding method yields the most parsimonious result. The additive binary coding method tends to force characters into ordered sequences of changes. Under unordered multistate coding the two or more mature states are free to transform in any sequence based on the most parsimonious arrangement. Additive binary coding is a logical assumption for unidirectional and progressive ontogenetic steps for certain characters like bone fusion events (e.g., partial fusion precedes full fusion) and bone textural changes (e.g., mottled texture precedes adult texture). However, other multistate character types are more ambiguous, where full-sized adults contain different character states with no clear indication of the sequence in which these develop (e.g., supraorbital horn shape). To use these features in the additive binary analysis, extra characters were added, so that the two (or more) non-nascent states are each independently scored as the most mature character state. By doing this, the two character states nullify one another, so that only the most parsimonious sequence of characters will be shared on a given internode of the tree. This type of coding was applied to the supraorbital region of the skull in order to test the hypothetical transition proposed by Ryan *et al.* (2001) that the supraorbital horns progress from pyramidal-shaped in juveniles, to inflated horns in more mature individuals, to resorbed masses in the most mature individuals. In the present analysis, the inflated horn character state and the resorbed mass character state are each individually considered the most mature state in two different characters.

To polarize the characters an all-zero hypothetical embryo was added to the data matrices. Specimens that duplicated identical codings for all characters, or all-zero coding, were removed from the data matrices in order to remove redundancy, because these specimens would, if included, group together and unnecessarily add to the data set. The resulting binary and multistate matrices were each executed in Phylogenetic Analysis Using Parsimony v. 4.0b (PAUP; Swofford, 1999) under a heuristic search. Because *C. apertus* growth cannot be observed firsthand, all characters were equally weighted and left unordered. A heuristic search was used because the sizes of the data sets were too large to efficiently run a branch and bound search with the available computing hardware. The results were then compared for consistencies between the two coding methods in order to find the most parsimonious tree for a given data set.

In the analysis of *Tyrannosaurus rex* by Carr & Williamson (2004), the authors included an artificial adult alongside the artificial embryo. This artificial adult was coded for the mature character states for each character in order to polarize the data away from the root and determine the most mature specimen. This additional step is required because the ontogram can be oriented so that any of the specimens above the artificial embryo are located directly opposite the root, which is the position of the most mature specimen. The addition of the artificial adult determines the axis of maturity by identifying the specimen that is recovered as the sister to the artificial adult. This provides an independent test in order to orient the tree correctly. The addition of this specimen may yield different results than the same data set without the artificial adult, which is why this technique is only used *a posteriori* as a heuristic method to determine the ontogenetic axis and hence the most mature specimen. In order to code this specimen the mature states were assumed to follow previous hypotheses of centrosaurine growth (Sampson, Ryan & Tanke, 1997; Ryan *et al.*, 2001).

The analyses were also run using both the ACCTRAN and DELTRAN character optimization methods. ACCTRAN optimizes character changes to occur as close to the root of the tree as possible, favoring reversals over convergent character changes farther up the tree. Conversely, DELTRAN optimizes character changes farther up the tree, selecting convergent character changes in lieu of reversals. These methods can therefore produce trees with similar specimen topologies, but different distributions of character changes. It is important to consider both optimization methods to compare character distributions for consistencies and inconsistencies between the two results, because both optimization types may yield a different relative order of development depending on the amount of missing data.

The robustness of the results was then tested with Bremer Decay and Bootstrap analyses. For the Bootstrap analysis 1000 replicates were executed using the results from the multistate character matrix in PAUP (Swofford, 1999). The binary matrix was excluded from the bootstrap analysis because it violates the assumption of character independence (Felsenstein, 1983). In the binary data set, characters are not independent because two (or more) characters are necessary to cover all character states for a given feature. If one of these characters is deleted, the corresponding character still remains and contributes to the bootstrap analysis.

In order to test the assumption that size is an acceptable proxy for ontogenetic status, a Spearman rank correlation was performed using the results of this cladistic analysis and frill length measurements. Frill length (rostrocaudal length along the midline) was used as a proxy for overall skull size because this portion of the skull is represented in most articulated specimens. Measurements were obtained from Sampson, Ryan & Tanke (1997) and supplemented with measurements for specimens not included in that study.

## Results

**Figure 2:**
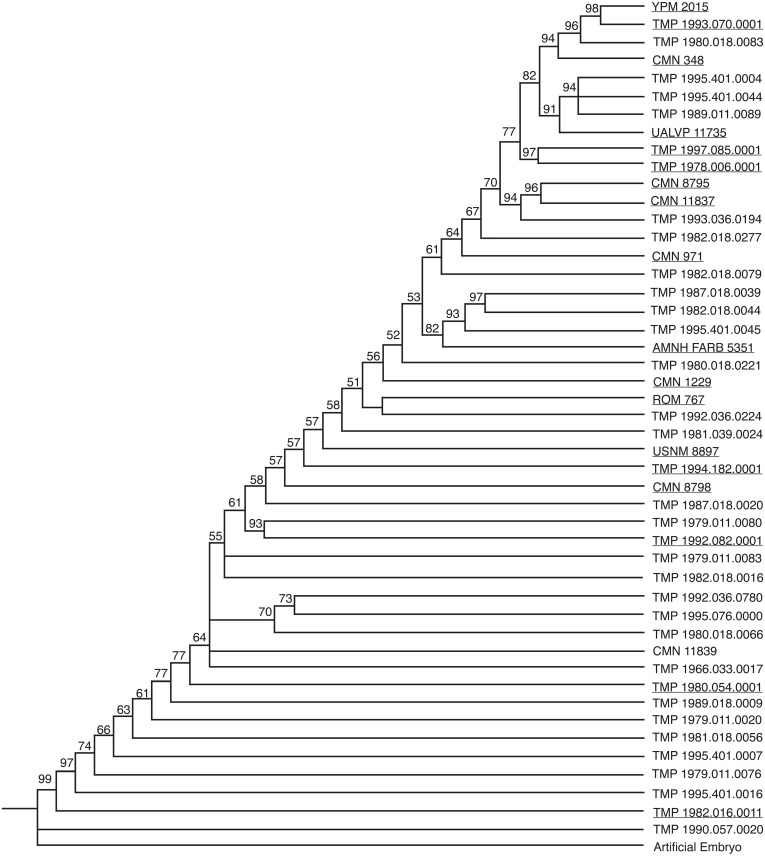
50% majority rule consensus tree for 100,000 MPTs, based on multistate character coding. This tree is 234 steps long, and has a CI of 0.5297. The numbers above each node represent the percent frequency of each grouping out of the 100,000 total trees. Underlined specimens are also found in the reduced analysis.

**Figure 3:**
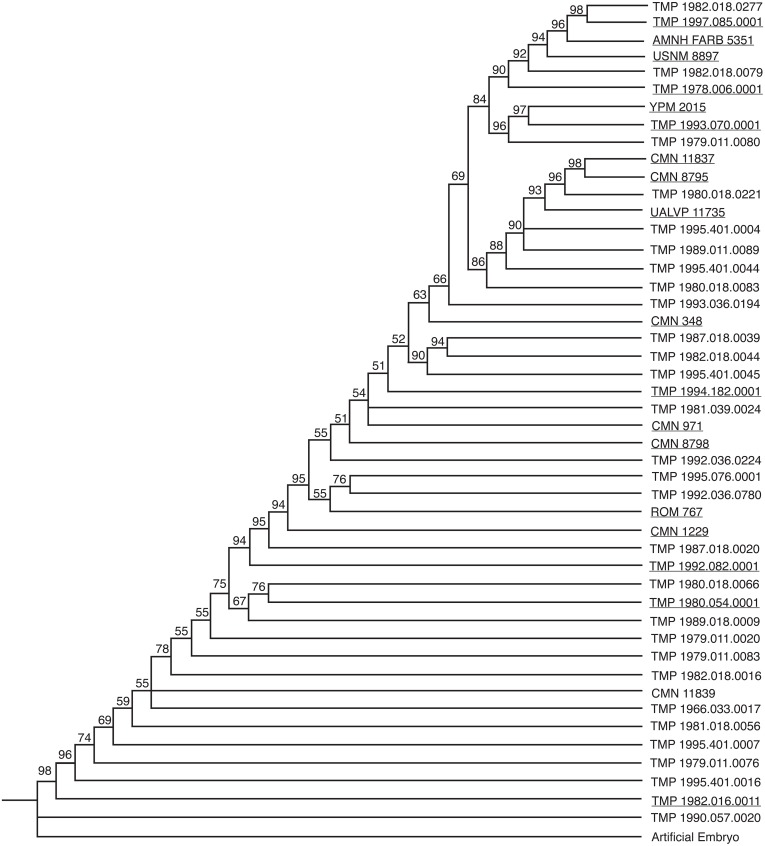
50% majority rule consensus tree for 100,000 MPTs, based on additive binary character coding. This tree is 265 steps long, has a CI of 0.4868. The numbers above each node represent the percent frequency of each grouping out of the 100,000 total trees. Underlined specimens are also found in the reduced analysis.

Two analyses of 47 specimens for 80 multistate characters and 130 additive binary characters independently yielded the maximum recoverable limit of 100,000 most parsimonious trees (MPTs) set in this analysis. The strict consensus tree, the semi-strict consensus tree, and the Adams consensus tree for both character types all independently collapsed into a single large polytomy; therefore a 50% majority rule consensus tree was obtained to recover structure in the data (Figs. 2 and 3). For both analyses, the resulting tree is linear, continuous, pectinate, and arranged with the least mature individuals near the base of the tree and progressively more mature individuals positioned away from the root.

The two coding methods generated trees that differ from one another. Both trees have areas of conflict, with three groups collapsing into polytomies in both data sets. The percent that each grouping is represented in the 100,000 saved trees (the number above the node in Figs. 2 and 3) is slightly higher for the binary data set, where groupings as a whole are supported on average in 78.8% of the trees, in contrast to 74.6% of the trees in the multistate data set. Conversely, the multistate tree has a higher consistency index (CI) (0.53) than the binary tree (0.49). The multistate tree is also shorter: 234 steps, in contrast to 265 steps in the binary tree. The majority rule consensus trees for both the binary and multistate analyses are topologically similar with many of the specimens occupying the same general position. Both analyses resulted in a consistent pattern for the six least mature specimens. Generally, the position of the more mature specimens is more variable between the two trees. The fragmentary specimens that are coded for fewer characters likely cause this disparity in ontogenetic rank between the binary and multistate analyses. For example, TMP 1979.011.0080, a disarticulated supraorbital horn containing part of the orbit, shows the greatest difference in ontogenetic rank between analyses (21.5 steps). In the multistate analysis this specimen is found as relatively immature, but in the binary analysis it is found with some of the relatively most mature individuals.

If isolated elements are ignored, articulated specimens share a very similar arrangement. A few articulated specimens, however, are recovered at different points on the tree. For example, in the multistate analysis ROM 767 is recovered as more mature than TMP 1994.182.0001, despite the fact that ROM 767 represents a smaller individual with shorter horns on the frill. This specimen is relatively less mature than TMP 1994.182.0001 in the binary analysis. The position of CMN 348 represents another inconsistency between the two analyses. In the binary tree this large skull is relatively less mature than in the multistate analysis. AMNH FARB 5351 shows similar disparity between analyses. In the multistate data set, this specimen is approximately midway between all of the other articulated skulls, whereas in the binary analysis it is the third most mature specimen. This specimen, although large, does not have as well developed ornamentations as many of the other skulls in this analysis, which suggests that this specimen is relatively less mature than in the binary tree.

The majority rule consensus trees for both analyses did not yield any unambiguous synontomorphies. These are characters that represent ontogenetically informative features that develop only once on the tree and are ‘inherited’ by all more mature specimens. Synontomorphies represent consistent growth patterns between all of the specimens in the analysis. The lack of synontomorphies in this analysis is likely the result of variability caused by the abundance of missing data in fragmentary and isolated specimens, which comprise the majority of the full data set.

**Figure 4:**
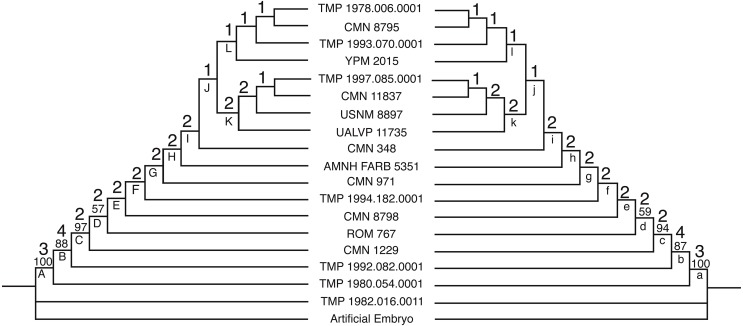
The single tree recovered for the reduced multistate data set. The tree is 206 steps long, with a CI of 0.5874. The tree on the left represents ACCTRAN optimization and the tree on the right represents DELTRAN optimization. The numbers above the nodes represent the Bremer Decay Index (upper) and the Bootstrap value (lower; <50% not shown). Synontomorphies for lettered nodes are listed in Table 1.

**Figure 5:**
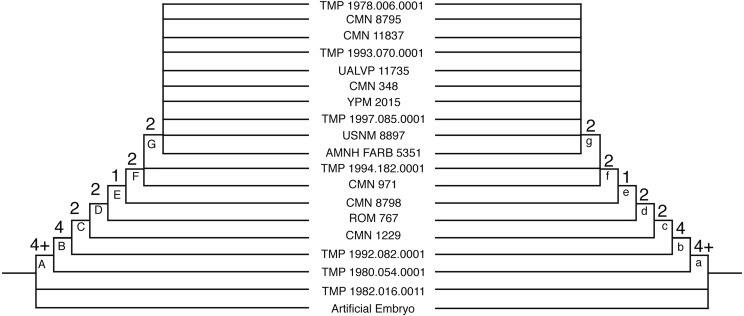
Strict consensus tree based the 13 MPTs recovered for the reduced binary data set. The tree is 236 steps long, with a CI of 0.5443. The tree on the left represents ACCTRAN optimization and the tree on the right represents DELTRAN optimization. The numbers above the nodes represent the Bremer Decay Index. Synontomorphies for lettered nodes are listed in Table 2.

In order to reduce conflict between the results and attempt to recover a better-resolved tree with unambiguous synontomorphies, the analysis was rerun with only partial and completely articulated skulls (*n* = 18) using a branch and bound search. The deletion of isolated elements reduced the variability caused by missing data and removed much of the ambiguity from the results, yielding fewer trees that are shorter and contain less internal conflict than the results from the complete data set. This supports the hypothesis that isolated material was the cause of the variability found in the complete analysis. The reduced multistate data set yielded a single most parsimonious tree of 206 steps with a CI of 0.59 (Fig. 4 and Table 1). The reduced binary data set yielded thirteen equally most parsimonious trees each with a length of 236 steps, a CI of 0.54 (Fig. 5 and Table 2). The overall topology of the strict consensus tree for the reduced binary data set is consistent with the single MPT from the reduced multistate data set, where the resolved ontogenetic progressions for the six least mature specimens is the same in both analyses.

**Table 1:** A list of synontomorphies for the reduced multistate analysis. The letters correlate with the designated nodes on Fig. 4, capitals are ACCTRAN optimization, lowercase are DELTRAN optimization.

Node	Character	Node	Character
A	Prefrontal suture with supraorbital unit partially obscured Palpebral suture with supraorbital unit partially obscured Supraorbital suture with jugal partially obscured Frontal midline suture partially obscured Adult texture on the premaxilla Non-striated texture on the nasal Mottled texture on the squamosal Rostral texture rugose Convexity on the rostrodorsal margin of the premaxilla present Rostrodorsal margin of the premaxilla rugose Steep ventral ridge on the premaxilla Expanded and rounded nasal horn Nasal horn suture completely obscured Deep frontal fontanelle Squamosal undulations laterally curved Thickened posterior margin between P2 and P3	a	Palpebral suture with supraorbital unit partially obscured Supraorbital suture with jugal partially obscured Squamosal posterolaterally concave and anteromedially convex Squamosal undulations laterally curved Thickened posterior margin between P2 and P3
B	Epiparietal four suture partially obscured Epiparietal five suture partially obscured Adult texture on the jugal Adult texture on the squamosal Lateral surface of the supraorbital horn rugose P2 horn present P1 horn oriented ventrally	b	Epiparietal four suture partially obscured Nasal horn suture completely obscured Adult texture on the jugal Adult texture on the squamosal P2 horn present P1 horn oriented ventrally Expanded and rounded nasal horn Convexity on the rostrodorsal margin of the premaxilla present Steep ventral ridge on the premaxilla Deep frontal fontanelle
C	Epiparietal six suture partially obscured Epiparietal seven suture partially obscured Straight nasal horn Constriction on the base of nasal horn present Large convexity on the jugal-maxilla border present Parietal fenestra over 50% sagittal length of frill	c	Parietal fenestra over 50% sagittal length of frill
D		d	Frontal midline suture partially obscured Rostral texture rugose Rostrodorsal margin of the premaxilla rugose Adult texture on the premaxilla Non-striated texture on the nasal Straight nasal horn Constriction on the base of nasal horn present Large convexity on the jugal-maxilla border present Lateral surface of the supraorbital horn rugose
E	Nasal suture rostral to nasal horn partially obscured Nasal suture caudal to nasal horn completely obscured Epiparietal four suture completely obscured Epiparietal five suture completely obscured	e	Nasal suture caudal to nasal horn completely obscured Epiparietal four suture fully obscured Epiparietal six suture partially obscured
F		f	Nasal suture rostral to nasal horn partially obscured
G	Nasal horn twice as long as base is wide Longitudinal grooves on the supraorbital unit present Sulci on the rostrodrosal margin of premaxilla present	g.	Epiparietal five suture completely obscured
H	Epiparietal six suture fully obscured	h	Nasal horn twice as long as base is wide Sulci on the rostrodrosal margin of premaxilla present
I	Procurved nasal horn	i	Epiparietal six suture fully obscured Epiparietal seven suture fully obscured Procurved nasal horn Longitudinal grooves on the supraorbital unit present
J	Nasal suture rostral to nasal horn fully obscured Episquamosal one suture partially obscured Episquamosal two suture partially obscured	j	Episquamosal two suture partially obscured
K	Episquamosal one suture fully obscured	k	Episquamosal one suture fully obscured
L	Prefrontal suture with supraorbital unit completely obscured Frontal midline suture fully obscured	l	Prefrontal suture with supraorbital unit completely obscured Frontal midline suture fully obscured

The Bremer Decay results for the reduced data sets indicate three steps or more of support for the position of the three least mature specimens. After these individuals, two steps support the majority of the groupings. In the reduced multistate analysis, the most mature specimens are only supported by a single step. This position is collapsed into a large polytomy in the reduced binary analysis. The Bootstrap analysis similarly recovered high values for the first three positions (100%, 88%, and 97% respectively). The position of the fourth most mature specimen was supported in only 57% of trees, followed by <50% for the three most mature. Both the Bremer Decay and the Bootstrap analyses recovered relatively low support for the trees. This indicates the presence of substantial variability within the data sets.

**Table 2:** A list of synontomorphies for the reduced binary analysis. The letters correlate with the designated nodes on Fig. 5, capitals are ACCTRAN optimization, lowercase are DELTRAN optimization.

Node	Character	Node	Character
A	Lacrimal suture with palpebral partially or fully obscured Prefrontal suture with supraorbital unit partially obscured Palpebral suture with the supraorbital unit completely or partially obscured Supraorbital suture with jugal partially or completely obscured Frontal midline suture partially or completely obscured Rostral texture rugose Mottled or adult texture on the premaxilla Non-striated texture on the nasal Mottled or adult texture on the squamosal Rostrodorsal margin of the premaxilla rugose Expanded and rounded nasal horn Supraorbital horn pyramidal shaped, inflated or remodeled One or two P1 horns Thickened posterior margin between P2 and P3 Convexity on the rostrodorsal margin of the premaxilla present Long triangular premaxillary process Steep ventral ridge on the premaxilla Deep frontal fontanelle Squamosal posterolaterally concave and anteromedially convex Squamosal undulations laterally curved Parietal sagittal length to width ratio 1.69 or less	a	Lacrimal suture with palpebral partially or fully obscured Prefrontal suture with supraorbital unit partially obscured Palpebral suture with the supraorbital unit completely or partially obscured Supraorbital suture with jugal partially or completely obscured Mottled or adult texture on the squamosal Supraorbital horn pyramidal shaped, inflated or remodeled One or two P1 horns Thickened posterior margin between P2 and P3 Squamosal posterolaterally concave and anteromedially convex Squamosal undulations laterally curved Parietal sagittal length to width ratio 1.69 or less
B	Nasal horn suture completely obscured at the tip, at least halfway, or entirely fused Nasal horn suture completely obscured at least halfway or entirely fused Jugal suture with lacrimal partially or completely obscured Epiparietal four suture partially or completely obscured Epiparietal five suture partially or completely obscured Adult texture on the squamosal Mottled or adult texture on the jugal Adult texture on the jugal Constriction on the base of nasal horn present Lateral surface of the supraorbital horn rugose P2 horn present P2 oriented medially or rostrally P1 oriented rostrally or ventrally	b	Nasal horn suture completely obscured at the tip, at least halfway, or entirely fused Nasal horn suture completely obscured at least halfway or entirely fused Jugal suture with lacrimal partially or completely obscured Epiparietal four suture partially or completely obscured Mottled or adult texture on the jugal Adult texture on the jugal Adult texture on the squamosal Lateral surface of the supraorbital horn rugose Long triangular premaxillary process Steep ventral ridge on the premaxilla Expanded and rounded nasal horn P2 horn present P2 oriented medially or rostrally P1 oriented rostrally or ventrally Deep frontal fontanelle Convexity on the rostrodorsal margin of the premaxilla present
C	Epiparietal six suture partially or completely obscured Epiparietal seven suture partially or completely obscured Straight or procurved nasal horn Large convexity on the jugal-maxilla border present Parietal fenestra over 50% sagittal length of frill P1 texture rugose or withered Cuplike depression between P1s slightly concave or concave	c	P1 texture rugose or withered Parietal fenestra over 50% sagittal length of frill P1 longer than wide or extends past the parietal fenestra
D	Shallow or deep caudal sulci on the P2 P1 longer than wide or extends past the parietal fenestra	d	Frontal midline suture partially or completely obscured Epiparietal five suture partially or completely obscured Rostral texture rugose Non-striated texture on the nasal Rostrodorsal margin of the premaxilla rugose Mottled or adult texture on the premaxilla Lateral surface of the supraorbital horn rugose Straight or procurved nasal horn Constriction on the base of nasal horn present Large convexity on the jugal-maxilla border present Shallow or deep caudal sulci on the P2 Cuplike depression between P1s slightly concave or concave
E	Nasal suture rostral to nasal horn partially or fully obscured Nasal suture caudal to nasal horn partially or fully obscured Nasal suture caudal to nasal horn fully obscured Epiparietal four suture completely obscured Epiparietal five suture completely obscured	e	Nasal suture caudal to nasal horn partially or fully obscured Nasal suture caudal to nasal horn fully obscured Epiparietal four suture completely obscured Epiparietal six suture partially or completely obscured
F		f	Nasal suture rostral to nasal horn partially or fully obscured Epiparietal five suture completely obscured
G	Sulci on the rostrodrosal margin of premaxilla present Nasal horn twice as long as base is wide Longitudinal grooves on the supraorbital unit present	g	Epiparietal seven suture partially or completely obscured Sulci on the rostrodrosal margin of premaxilla present Nasal horn twice as long as base is wide Longitudinal grooves on the supraorbital unit present

To test the use of large size differences as justification for identifying juvenile character states, a Spearman rank correlation (Table 3) was executed using the reduced multistate data set, because this was the only fully resolved tree. The split near the top of the reduced multistate tree (Fig. 4) is interpreted here as an oversampling of a single growth stage (see Discussion section), and specimens in the group with less cumulative growth changes were all assigned the same ontogenetic rank. The results of this analysis yielded a strong (0.952) congruence between ontogenetic rank and frill length.

**Table 3:** Spearman rank correlation between frill length and ontogenetic rank.

Specimen	Ontogenetic rank	Frill length (mm)	Length rank
TMP 1982.016.0011	1	225	1
TMP 1980.054.0001	2	575	2
TMP 1992.082.0001	3	587^*^	4
ROM 767	4	580	3
CMN 8798	5	590	5
TMP 1994.182.0001	6	645^*^	7
CMN 971	7	620	6
AMNH FARB 5351	8	679	9
UALVP 11735	9.5	656	8
TMP 1997.085.0001	9.5	695^*^	11
CMN 8795	11	688	10
D = 0, 0, 1, 1, 0, 1, 1, 1, 1.5, 1.5, 1			
D^2^ = 0, 0, 1, 1, 0, 1, 1, 1, 2.25, 2.25, 1 = 10.5			
P = 1 − ((6 ∗ 10.5)∕11(11^2^ − 1)) = 0.952			

* Measurements not from Sampson, Ryan & Tanke (1997).

### Addition of an artificial adult

An artificial adult was added *a posteriori* to the reduced multistate and binary data sets in order to identify the most mature specimen. In the multistate analysis, the addition of the artificial adult added one step to the analysis (207 steps) and, as with the original data set, a single MPT was recovered. The artificial adult was recovered as the sister specimen to YPM 2015 above node L (Fig. 4). The reduced binary analysis also recovered the artificial adult as the sister to YPM 2015, and the addition of the artificial adult added six steps. This analysis yielded four MPTs and the strict consensus was better resolved than the binary analysis without the artificial adult. The arrangement of specimens is not in conflict with the results yielded without the artificial adult.

### Growth stages

**Figure 6:**
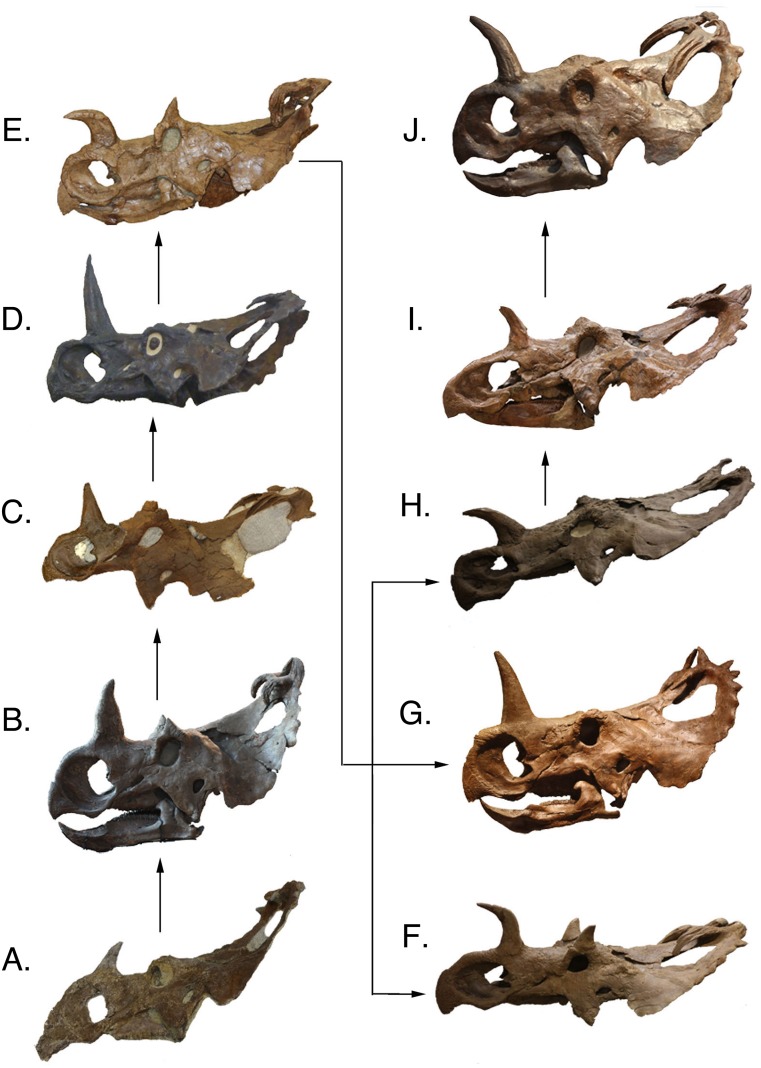
Complete skulls in lateral view arranged in ontogenetic order from the relatively least mature to the relatively most mature specimens, based on the reduced multistate tree. Skulls are not to scale. (A) TMP 1992.082.0001; (B) ROM 767; (C) TMP 1994.182.0001, (D) AMNH FARB 5351, (E) CMN 348; (F) UALVP 11735; (G) USNM 8897; (H) TMP 1997.085.0001; (I) CMN 8795; (J) YPM 2015. Images of TMP 1994.182.0001, AMNH FARB 5351, and CMN 8795 are reversed (mirrored). AMNH FARB 5351 and TMP 1997.085.0001 are represented here by casts. Division of Vertebrate Paleontology, YPM 2015. Courtesy of the Peabody Museum of Natural History, Yale University, New Haven, Connecticut, USA.

The following is the hypothesized ontogenetic progression for *C. apertus* as reconstructed based on the analysis of the reduced data set of reasonably complete and associated cranial material (Figs. 4, 5 and 6 for complete cranial material). All character changes presented below occur in the same position on fully resolved portions of the trees for both the multistate character and additive binary character data sets. The character changes are discussed according to their positions under ACCTRAN optimization. The character changes discussed here are recovered as synontomorphies under both ACCTRAN and DELTRAN optimization; synontomorphies that are found under only one optimization type are excluded. Those character changes potentially represent individually variable characters that are not ‘inherited’ through the ontogenetic sequence because they develop on the terminal branches and not the stem of the tree (full list of character changes found in Tables 1 and 2). The use of the term growth stage follows Carr & Williamson (2004), where each stage occurs above a resolved node and is exemplified by the specimen(s) branching from it.

The first growth stage is represented by the presence of juvenile growth characters. The exemplar is TMP 1982.016.0011, a fragmentary individual that could only be coded for the parietal and squamosals. This specimen was coded almost entirely with nascent growth states and shares a position with the artificial embryo. This specimen contains no synontomorphies and is also the smallest specimen in this analysis. Because TMP 1982.016.0011 possessed few codeable elements, the character states that define this growth stage are largely reconstructed from the hypothetical anatomy of the artificial embryo. In this growth stage, all cranial sutures remain unfused, all bones possess striated bone texture, the frill is relatively featureless, the nasal horn is recurved, and the supraorbital horns are short and sharp. The conditions seen in the first growth stage of *C. apertus* are also the seen in the type specimen of *Brachyceratops* (USNM 7951), indicating the juvenile status of this specimen, and providing further evidence for the invalidity of the genus *Brachyceratops* (McDonald, 2011).

The second growth stage (Node A; e.g., TMP 1980.054.0001) is supported by eleven synontomorphies. In this growth stage, suture closure is partial between the palpebral and supraorbital unit, between the prefrontal and the supraorbital unit, between the jugal and the supraorbital unit, and along the frontal midline. Bone texture is adult on the premaxilla, mottled on the squamosal, and non-striated (adult or mottled) on the nasal. The nasal horn is wide and rounded in cross section, and the undulations on the squamosal extend laterally rather than caudally. Finally, the rostral bone has a rugose texture, a convexity is present on the rostrodorsal margin of the premaxilla, a steep angle is seen on the ventral ridge of the premaxilla, the frontal fontanelle is deep, and the caudal margin of the frill is thick. Growth stage two represents the greatest abundance of character changes for the growth series. This can be partially attributed to the presentation of ACCTRAN over DELTRAN character coding in this summary. This abundance of synontomorphies may also be a result of missing data, as TMP 1980.054.0001 represents a much larger specimen than TMP 1982.016.0011, meaning it is likely that growth stages are missing between stage one and stage two.

The third growth stage (node B; e.g., TMP 1992.082.0001) is supported by seven synontomorphies. In this growth stage suture closure is partial for epiparietals four and five. Adult texture is seen on the squamosal and the jugal, as well as rugose texture on the lateral surface of the supraorbital horn. Changes in cranial ornamentations include the development of P2 and a ventrally oriented P1. This growth stage represents the first stage at which a specimen possesses the diagnostic characteristics of *Centrosaurus apertus*. Prior to this stage, the parietal processes are small and uncurved. Individuals at the first and second growth stages look no different than other juvenile centrosaurines (Ryan *et al.*, 2001; Sampson, Ryan & Tanke, 1997) and without stratigraphic evidence these specimens would not be identifiable to *C. apertus*.

The fourth growth stage (node C; e.g., CMN 1229) is supported by six synontomorphies. In this stage, suture closure is partial for epiparietals six and seven. This indicates that at stage four all epiparietals will demonstrate some degree of fusion. At this stage, the cranial ornamentation changes so that the nasal horn is straight. Other growth characters include development of a convexity on the jugal-maxilla border, and expansion of the parietal fenestra in all directions.

The fifth growth stage (node D; e.g., ROM 767) is supported by no synontomorphies in the ACCTRAN analysis. In the DELTRAN analysis node D is supported by nine synontomorphies. The differences between the character distributions for node D in the ACCTRAN and DELTRAN optimized analyses represent different hypotheses for growth characters, based on missing data in the surrounding specimens. CMN 1229 (node C) is an isolated parietal. Using ACCTRAN optimization, all non-parietal character changes seen in ROM 767 (node D) and not in relatively less mature specimens will be associated with CMN 1229 (node C).

The sixth growth stage (node E; e.g., CMN 8798) is supported by four synontomorphies. Suture closure is partial on the rostral margin of the nasal, complete on the caudal margin of the nasal, and complete for epiparietals four and five. After this point the tree produced by additive binary characters collapses into a polytomy containing TMP 1994.182.0001, CMN 971, and another polytomy containing the remaining mature specimens (Fig. 5).

In the multistate analysis, the seventh growth stage (node F; e.g., TMP 1994.182.0001) is not supported by any unequivocal synontomorphies. This result, like in stage five, is produced by the differences in ACCTRAN and DELTRAN optimization. In the DELTRAN analysis the partial fusion of the intranasal suture on the rostral margin of the nasal is delayed until this growth stage.

The eighth growth stage (node G; CMN 971) is supported by three synontomorphies. The nasal horn is twice as long as it is wide, reaching its maximum dorsoventral height for this structure at approximately this stage of development. Further synontomorphies include the development of sulci on the rostrodorsal margin of the premaxilla, and on the supraorbital unit, creating well-defined longitudinal grooves on its medial surface. This increased vascularization, in association with deep sulci that are developed on P1s of CMN 971 and variably in other less mature individuals, may represent a precursor to future remodeling of the ornamentation.

The ninth growth stage (node H; e.g., AMNH FARB 5351) is supported by a single synontomorphy, the complete fusion of epiparietal six. The epiparietals show a consistent pattern to the order of fusion in a caudal to rostral direction. Fusion begins with the partial fusion of epiparietals four and five in stage three and progresses with the partial fusion of epiparietals six and seven at stage four. At stage six epiparietal four and five become fully fused, and at stage nine epiparietal six becomes fully fused as well. This indicates that not only do the epiparietals begin to fuse in a caudal to rostral direction, but also completely fuse in this same order. This implies that the duration of fusion for each epiparietal is subequal across the frill.

The tenth growth stage (node I; e.g., CMN 348) is supported by a single synontomorphy, a procurved nasal horn. The nasal horn in CMN 348 is curved so that the distal end is roughly parallel to the ground. This adult condition is also seen in *Einiosaurus procurvicornis*, but to a greater extent where some of the individuals possess a horn that extends rostroventrally (Sampson, 1995).

The remaining most-mature specimens are split into two separate growth series (node J; Fig. 4). One of the series (node K) is united by a single synontomorphy, the complete fusion of episquamosal one. The other series (node L), containing YPM 2015, is supported by two synontomorphies: the fusion of the frontal midline suture and the obliteration of the prefrontal suture with the supraorbital unit. The growth series at node L represents the most mature individuals in the analysis because they contain the most cumulative character changes. This result is consistent with the interpretation that YPM 2015 is the most mature specimen as determined by addition of the artificial adult.

## Discussion

### Comparison of additive binary and multistate coding methods

Historically, character coding has been a widely discussed topic in the field of phylogenetics (e.g., O’Grady & Deets, 1987; Pleijel, 1995). Multistate characters are often transformed into additive binary characters, where a single multistate character is expressed as more than one binary character. Although this practice is widely used, it has been criticized for unjustifiably weighting certain areas of the data set (O’Grady & Deets, 1987). For ontogenetic studies of growth, both coding styles are prevalent in the published literature with no formal consensus on which is the optimum coding method (Brochu, 1996; Carr & Williamson, 2004; Carr, 2010; Longrich & Field, 2012).

In order to determine the most parsimonious result, the trees are compared based on their lengths. Even though both coding methods have a different number of characters (for example, it takes two binary characters to cover a single multistate character with three states), both coding styles will retain (approximately) the same number of character changes and would hypothetically yield a tree of (approximately) the same length. Further, the CI reduces any inconsistencies by dividing the theoretical minimum number of steps by the observed number of steps in the tree.

This study analyzed the same specimens of *Centrosaurus apertus* using both the multistate and additive binary coding methods. In total, 80 multistate characters were expanded into 130 binary characters. In both the complete and reduced data sets the use of multistate characters resulted in a tree that was approximately 10% shorter than the tree recovered using binary characters. Furthermore, the multistate trees have a higher CI than that of the binary trees. The single tree recovered for the multistate analysis of the reduced data set represents the shortest tree recovered under all coding combinations for this study. In contrast, the binary analysis of the reduced data set recovered thirteen MPTs that are 30 steps longer. This indicates that multistate character coding represents the most efficient method for recovering a hierarchical pattern from this data set.

### Growth series bifurcation

Near the top of the complete binary analysis and the reduced multistate analysis (Figs. 3 and 4) is a split among the most mature specimens. This split creates two separate groups that include their own growth series. This split can be interpreted in multiple ways: (1) one group represents an oversampling of a single stage of growth and the other group represents the continued growth series, (2) the split represents sexual dimorphism where one group represents males and the other group females, (3) the groupings represent different species that are only morphologically different as adults, and (4) the split represents uneven ontogenetic character acquisition (individual variability) in late ontogeny.

If the split represents sexual dimorphism, two requirements should be met. First, morphological differences between the two groups should exist, which would hypothetically represent structures used for sexual display. Second, a shared sequence of growth changes should exist that both sexes develop independently. Although both groups share a reduction in size of the ornamentations in the most mature specimens, there are no sexual display structures uniquely attributable to one group or the other. There is also a lack of shared growth characters between the two groupings. Furthermore, the order and placement of specimens within the two groups is inconsistent between the complete binary and reduced multistate analyses. This makes both the sexual dimorphism and the different species hypotheses unlikely.

Individual variability during ontogenetic character acquisition is an intriguing idea, and would explain the difficulty of producing a consensus between the two reduced analyses for the most mature individuals. However, it is unclear how a bifurcation could be the result of variability without the presence of character dimorphism or shared growth characters. Because of this doubt, oversampling of a single growth stage (hypothesis 1 above) is tentatively the best explanation for this split, where the lineage in the multistate analysis following Node L (Fig. 4) represents the most mature specimens.

### *Centrosaurus* Ontogeny

#### Nasal

The nasal horn of centrosaurines represents one of the most spectacular cranial structures in all of Dinosauria. Throughout ontogeny, this structure goes through dramatic changes in shape that continue well after adult size is attained. In juveniles, the nasal horn begins as a small triangular structure formed by the fusion of the right and left nasals. The smallest horns are mediolaterally narrow with a recurved tip. With growth the nasal horn enlarges and changes orientation, becoming straight and erect. In this analysis, specimens close to the transition from a recurved nasal horn to straight nasal horn show a transitional morphology where the basal part of the horn is straight and only the dorsal tip of the horn is recurved, as present in ROM 767. The straight nasals fuse in a dorsal to ventral direction, and then continue to fuse rostrally and caudally from the nasal horn. Ryan *et al.* (2001) document many unfused rostral sutures in the largest horncores collected from Bonebed 43 in Dinosaur Provincial Park. This is consistent with the results of this analysis, where some of the large disarticulated nasals are recovered in the tree as less mature than smaller articulated specimens that possess a closed suture.

The largest individuals show more variation in nasal horn orientation than the smaller ones. Full-sized adult specimens possess nasal horns that are either straight or procurved, with some of these individuals possessing a deep basal constriction. Ryan *et al.* (2001) hypothesized that the degree of curvature and basal constriction are both individually variable conditions that develop in large, presumably mature, individuals.

**Figure 7:**
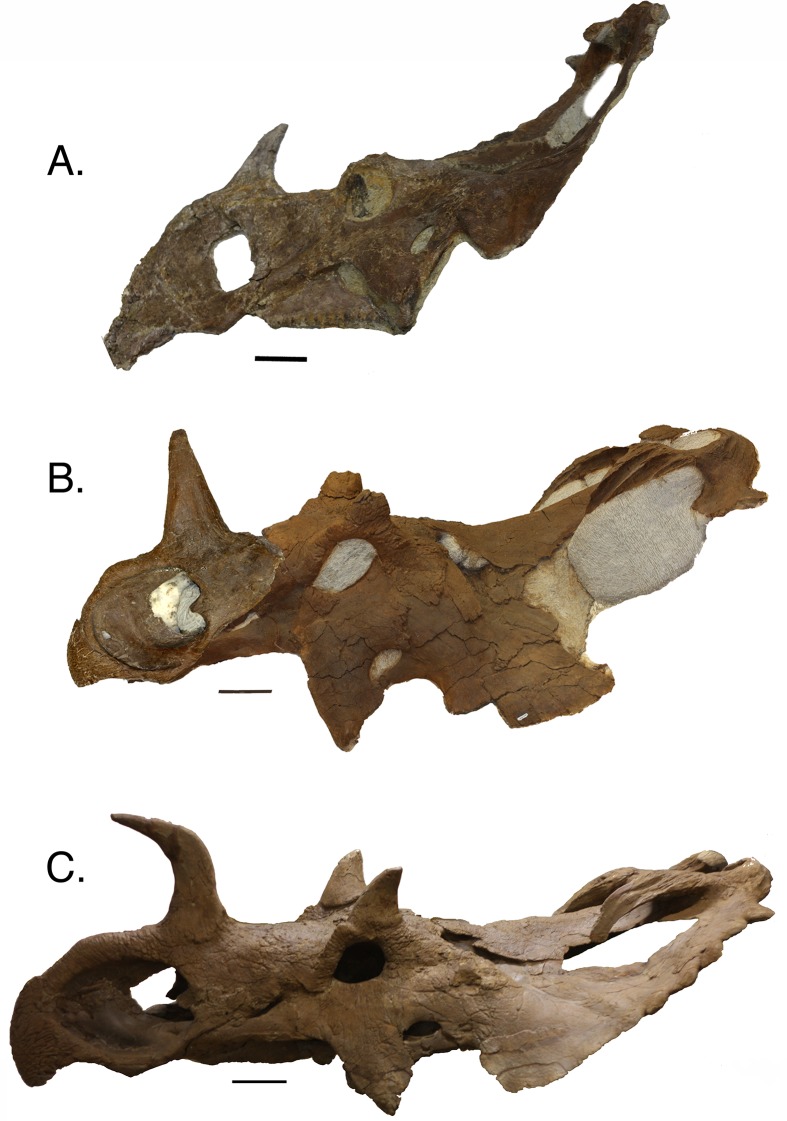
Three nasal horn character states. (A) recurved, TMP 1992.082.0001; (B) straight, TMP 1994.182.0001 (composite, mirror imaged); (C) procurved, UALVP 11735. Scale is equal to 10 cm.

This analysis recovers nasal horn shape as an ontogenetic characteristic, where straight horns are found in relatively less mature individuals, in contrast to procurved horns, which are found in the most mature individuals (Fig. 7). This progression was recovered in the multistate trees of both the complete and reduced data sets, and is consistent with the reduced additive binary tree. In the binary tree for the complete data set, a straight-horned specimen (AMNH FARB 5351) was recovered as relatively more mature than some of the procurved specimens (e.g., CMN 348). Other disarticulated straight nasal horns are all recovered relatively less mature than the procurved specimens, indicating that AMNH FARB 5351 is the only exception to this transition. Some individuals, such as CMN 348, possess a deep groove along the caudal surface of the nasal horn. This feature is variable among specimens and does not show an ontogenetic pattern. Similarly, the degree of basal constriction on the horn shows no pattern.

There is precedent for this type of transition elsewhere in Ceratopsidae. A similar ontogenetic change in horn orientation occurs in the supraorbital region of *Triceratops*. Throughout ontogeny, the supraorbital horns of *Triceratops* change from recurved in juveniles, to straight and rostrodorsally directed in relatively more mature individuals, to bent and rostrally directed in some of the largest individuals (Horner & Goodwin, 2006). Although the morphological progression in *Centrosaurus* and *Triceratops* are likely independently evolved, this demonstrates that complex changes in ornamentations are also present throughout the growth series of other ceratopsids.

### Supraorbital unit

Previous studies have documented that the smallest known centrosaurine specimens possess small horns as outgrowths of the postorbital (Sampson, Ryan & Tanke, 1997; Ryan *et al.*, 2001). With maturity these horns become tall and pyramid shaped, as seen in TMP 1980.054.0001. In larger individuals the supraorbital horns have a variety of morphologies. The largest articulated skulls generally exhibit one of two supraorbital shapes: a tall and rounded horn, as seen in UALVP 11735, or a low rugose mass, as in AMNH FARB 5351. Other individuals demonstrate intermediate morphologies. Ryan *et al.* (2001) explained these morphological differences as a result of remodeling, where the triangular horns in juveniles are resorbed producing pitting on the apex. In this analysis, the two conditions appear to possess wide individual variation in specimens of similar relative maturity. For example, TMP 1992.082.0001 is a relatively immature specimen that possesses a low rugose mass over the orbit, but a more mature specimen, ROM 767, has a tall and sharp horn (Fig. 6). In general, the most mature specimens demonstrate the low rugose mass, but exceptions are seen, most notably UALVP 11735 (Fig. 6). As previously hypothesized (Sampson, Ryan & Tanke, 1997; Ryan *et al.*, 2001), this dramatic shape change is the result of supraorbital pitting, which remodels this region from the horn-like juvenile condition to the rugose mass that is seen in many adults. Supraorbital pitting is present in most of the large skulls; however, variability is common. YPM 2015 is the most mature specimen recovered in the analyses and it has less pitting than some relatively less mature specimens. The inconsistency of pitting in the most mature specimens may represent sexual or individual variation of this particular feature within the sample of *C. apertus*.

Ryan *et al.* (2001) designated three growth stages for supraorbital specimens from Bonebed 43. Stage one contains specimens with unfused bones and small-pyramid shaped horns with non-rugose texture. The second growth stage contains slightly more robust specimens with a postorbital fused to all surrounding bones. In the final growth stage all bones fuse into the supraorbital unit and the horn becomes rugose and pitted. These stages do not correlate directly with distinct size classes, as size in this sample set is continuous. The present analysis recovers a similar progression of growth changes in the data set; however, stage three appears relatively early in maturity. By the maturity level of TMP 1994.182.0001 (Node F of Fig. 4), the supraorbital horn is inflated, resorption pits have developed, the individual supraorbital bones have begun to fuse, and the external texture of the horn is rugose. In the reduced multistate analysis this specimen is recovered as relatively immature (growth stage seven) before many of the major cranial changes occur (e.g., procurved nasal horn). This demonstrates that the supraorbital unit growth stages of Ryan *et al.* (2001) cannot be used to determine the ontogenetic status of relatively mature individuals.

### Frill

**Figure 8:**
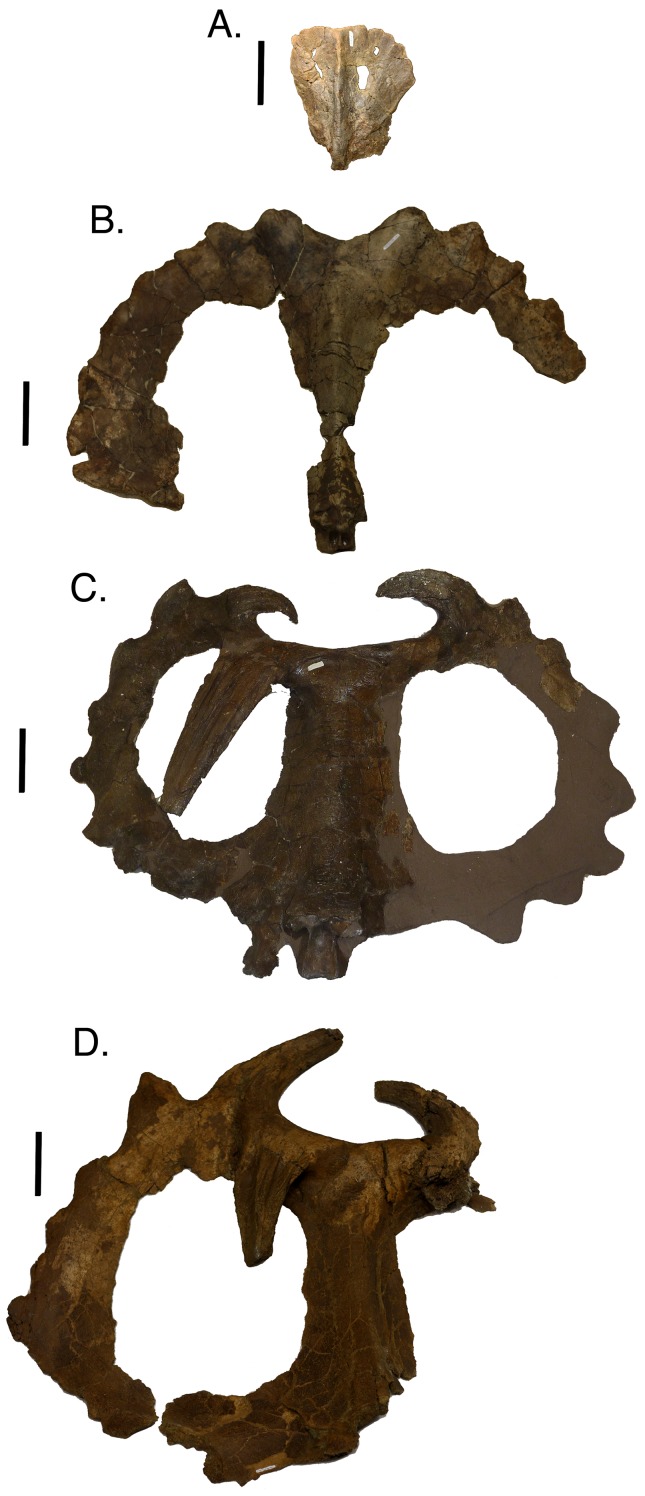
Parietals in dorsal view arranged in an ontogenetic progression from top to bottom based on the reduced multistate analysis. (A) TMP 1982.016.0011; (B) TMP 1980.054.0001; (C) CMN 971; (D) TMP 1978.06.0001. Scale is equal to 10 cm.

The parietal-squamosal frill (Fig. 8) is one of the most diagnostic structures in centrosaurines. The horns and hooks that surround the frill are often distinctive to the level of genus or species and the uniqueness of this arrangement may have served a purpose in intraspecific recognition (Sampson, 1995; Padian & Horner, 2010). In adult *C. apertus* the most medial horn (P1) curves rostrally, occasionally extending more than halfway down the frill. The second horn (P2) curves medially, and the variably developed third horn (P3) extends caudally (Fig. 8).

**Figure 9:**
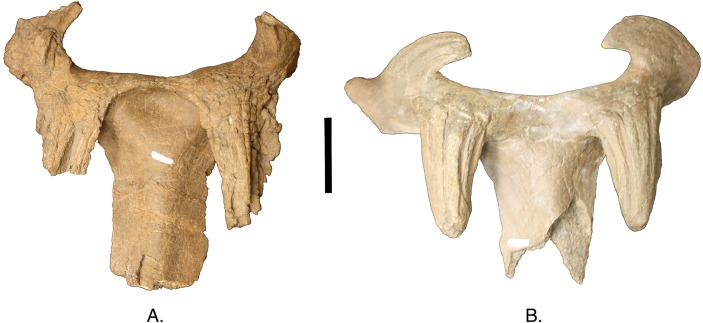
Dorsal view of parietals demonstrating the withered (A; TMP 1982.018.0277) and non-withered (B; TMP1982.018.0790) character states. Scale is equal to 10 cm.

In small individuals in the first growth stage, such as TMP 1982.016.0011, the diagnostic horns and hooks have not yet developed along the frill margin and the parietal fenestra is small (Fig. 8). In the next more mature skull, TMP 1980.054.0001, P1 and P2 have developed into well-defined bumps that extend caudally from the frill margin, and the parietal fenestra has expanded to remove a large portion of the interior of the frill (Fig. 8). In relatively more mature specimens (TMP 1992.182.0001; growth stage three), P1 changes orientation and curls rostroventrally as P2 extends further caudally (Fig. 8). With greater maturity P1 continues to elongate ventrally so that it overlays the parietal fenestra and P2 begins to curve medially. TMP 1994.182.0001 and CMN 971 (growth stage seven or greater) exemplify further changes that occur with maturity, where deep longitudinal sulci develop on P1 and P2 horns (Fig. 8). At this level of maturity P1 may extend rostroventrally more than halfway down the frill and P3 may develop into an elongated horn. The longitudinal sulci may eventually expand, giving P1 and P2 a withered appearance, as seen in TMP 1978.006.0001 (Fig. 8) and TMP 1982.018.0277 (Fig. 9).

Many of the most mature specimens (CMN 8795 and TMP 1997.085.0001) have greatly shortened P1s (Fig. 6). This reduction may be a result of the continual extension of the longitudinal sulci seen in less mature individuals. This decline in horn size in the most mature specimens is similar to the reduction seen in the supraorbital region. All known specimens with reduced P1s also possess resorbed supraorbital horns (CMN 8795 and TMP 1997.085.0001). Furthermore, the nasal horn in CMN 8795 is also reduced in length and width compared to other similarly sized specimens. This indicates that there is a complete reduction in ornamentations associated with late maturity.

This reduction in ornamentation size with increasing maturity is not unique to *C. apertus*. The derived chasmosaurine *Triceratops* similarly reduces the size and shape of ornamentations (Horner & Goodwin, 2006). In less mature animals, the epiparietals and episquamosals are tall and triangular. In mature individuals, these are low and elongate, and become difficult to recognize in some of the largest skulls (Horner & Goodwin, 2006). Ontogenetically linked ornamental reduction is also seen in *Pachycephalosaurus* (Horner & Goodwin, 2009) and *Tyrannosaurus rex* (T. Carr, personal communication, 2013). The widespread nature of this phenomenon throughout Dinosauria suggests that the reduction of cranial ornamentations with increasing levels of maturity is a primitive (plesiomorphic) feature for the clade.

Larger *Centrosaurus* skulls, including the *C. apertus* holotype (CMN 971; Fig. 8), occasionally have only a single P1 horn on either the left or right side of the frill. Skulls with this condition show no signs of taphonomic modification or pathology, indicating a complete absence in the growth of the contralateral P1 horn. In this analysis, skulls with a single P1 horn are found at multiple growth stages, signifying no ontogenetic link to the asymmetrical P1 horns. The best explanation for this condition is that the possession of a single P1 horn is a variable trait within the population, where the second horn does not begin to develop in these individuals.

Sampson, Ryan & Tanke (1997) hypothesized that the epiparietals and episquamosals fuse in a caudal to rostral sequence. The results of this study are consistent with that hypothesis, where epiparietal four and five fuse relatively early, before the fusion of epiparietal six and seven. In the reduced analysis of multistate characters, episquamosals fully fuse later in maturity than epiparietals; this area is unresolved in the reduced binary analysis. The episquamosals tend to be much smaller and do not appear to preserve as readily as the epiparietals. The small size of the episquamosals most likely made these structures more susceptible to taphonomic modification, which explains their absence in many large specimens.

### Proxies for growth

The quantitative results from the three principal maturity indicators (bone fusion, bone texture, and ornamentation) are shown in Tables 4 and 5. For the reduced multistate analysis, bone fusion characters contained the highest cumulative CI, indicating that these are the least variable characters, as they tend to change states comparatively fewer times than the other proxies. In this analysis, bone fusion can be broken down into two types: skull bones and epiparietals/episquamosals. If fusion characters for the latter are removed, the average consistency declines to below the cumulative CI for bone textural characters, which is the second best proxy for growth. This indicates that epiparietal and episquamosal fusion is a reliable ontogenetic indicator in this species, and is consistent with the findings of Sampson, Ryan & Tanke (1997).

**Table 4:** The cumulative CI for different regions of the skull.

Region	Bone	Number of characters	Cumulative CI for bone	Cumulative CI for region
Nasal	Nasal	11	0.864	0.864
Supraorbital	Unit (lacrimal, palpebral, prefrontal, postorbital)	11	0.709	0.709
Frill	Squamosal	11	0.765	0.683
	Parietal	23	0.644	
Frontal	Frontal	4	0.792	0.792
Other	Rostral	3	0.667	0.710
	Premaxilla	9	0.769	
	Maxilla	3	0.864	
	Jugal	5	0.713	
Total		80		

**Table 5:** The cumulative CI for the different proxies for growth.

Character type	Number of characters	Cumulative CI
Bone texture	6	0.833
Bone fusion	21	0.860
Bone fusion (excluding epiparietals/episquamosals)	13	0.813
Display structures	12	0.618
Other	41	0.643

Although this analysis recovers bone fusion as a relatively reliable indicator of ontogenetic stage in *Centrosaurus apertus*, many of the largest skulls retain open sutures (e.g., CMN 348). Bone fusion has been suggested as an unreliable proxy for growth in other ceratopsians. Brown & Schlaikjer (1940) showed that bone fusion is inconsistent in the basal neoceratopsian *Protoceratops andrewsi*, where some of the largest individuals retain open sutures and many of the smaller ones possess fully fused sutures. Also, bone fusion in the chasmosaurines *Triceratops* and *Torosaurus* appear variable, with some of the largest skulls possessing incompletely fused cranial bones (Scannella & Horner, 2011; Longrich & Field, 2012). The lack of consistency for bone fusion characters, especially if epiparietal and episquamosal characters are ignored, supports these previous observations and suggests that bone fusion should not be relied upon as the sole criterion when determining relative maturity in horned dinosaurs.

The second best proxy for growth is bone textural changes. Bone textural changes have been shown to be reliable growth proxies in horned dinosaurs, especially in the frill (Sampson, Ryan & Tanke, 1997; Ryan *et al.*, 2001; Brown, Russell & Ryan, 2009; Tumarkin-Deratzian, 2010). In this analysis, bone textural changes were extremely effective at identifying relative ontogenetic stage in the least mature specimens. In all of the full-sized specimens, adult texture covered large portions of the cranial bones, rendering this proxy useless in determining relative maturity among the largest individuals.

The least effective proxies for growth in this analysis are the characters that are based on cranial ornamentations. The morphology of horns, especially in the supraorbital region, is highly variable between specimens, and within a single individual. It is likely that the development of these structures is controlled by more factors than relative maturity alone (e.g., sex, nutrition, status within a social hierarchy, etc.). Further, the cranial ornamentations reduce in size in the most mature individuals, giving their skulls less distinctive ornamentations than those seen in relatively immature specimens. Although some of these ornamentations (e.g., P1) show a fairly consistent pattern of growth, it does not follow an expected sequence where increasing maturity equates to more elaborate ornamentations. This pattern of development limits the utility of the ornamentations alone as ontogenetic indicators, as the immature and relatively most mature individuals both tend to have small ornamentations, which could hypothetically look similar if a specimen is taphonomically modified or pathological.

No single growth character type can be used to determine the relative maturity of an individual *C. apertus* specimen throughout the entire growth series, but the combination of bone textural changes in tandem with nasal horn curvature and epiparietal/episquamosal fusion appear to be the best proxies for growth in this taxon.

### Size as an ontogenetic indicator

Based on the results of the Spearman rank correlation (Table 3) frill length in *C. apertus* generally increases with ontogenetic stage, where the smallest articulated frill measures 225 mm in length and the largest 695 mm. The five least mature specimens demonstrate superior correlation of size with relative maturity than the six most mature specimens. Only two of the least mature specimens do not represent the same ontogenetic rank as frill size rank. Conversely, all six of the largest skulls differ in ontogenetic rank and frill size rank; however, this difference is no greater than 1.5 steps. Therefore, this analysis shows a strong positive correlation between ontogenetic stage and frill length. Increases in size, however small, continue throughout the ontogenetic progression of *C. apertus*. Caution should be taken when using this proxy, because it is insufficient to distinguish ontogenetic status between two similarly sized animals.

The results of the Spearman rank correlation also suggest that large-scale sexual size dimorphism is not present in *C. apertus*. The strong congruent signal obtained shows a positive relationship between frill size and ontogenetic status, with no specimen exceeding the next by more than 25 mm in length. If sexual size differences were present, it would be assumed that many of the most mature specimens from the smaller sex would have a size rank far below less mature individuals of the larger sex. The relatively uniform increase in size and ontogenetic rank implies that both sexes were roughly the same size throughout growth. This result needs to be tested further using a larger data set, making the likelihood of sampling both sexes greater.

### Histology

Histology is a growing science in the field of dinosaur paleontology (Horner, De Ricqlès & Padian, 2000, etc.). These studies have yielded bountiful data with regard to dinosaur ontogeny, including important information on the approximate ages of individual specimens (e.g., Erickson & Druckenmiller, 2011). Histological studies of *C. apertus* have not been widely published; moreover with few complete specimens it is difficult to translate ages derived from long bone histology to ontogenetic changes that occur in the cranium (Tumarkin-Deratzian, 2010). However, when these data are recovered it will provide further testing to the hypothesis provided here (for example, bone remodeling should be present in all of the most mature specimens recovered in this analyses). In addition to understanding size changes, approximate numerical ages will give further insight into when the growth stages presented in this analysis occur. Caution must be advised, however, as numerical age and maturity are not always synonymous.

Another possibility includes the addition of histological data into a cladistic analysis. Histology is another proxy for growth, and could be incorporated into this type of analysis to test its consistency with other growth characters. If histological data prove more reliable than other growth proxies, it should provide more resolved and robust trees that can be used to identify other sources of variability. This holistic approach is strongly advised for future ontogenetic studies.

## Conclusions

 The principal conclusions of this study are as follows:

(1) A hypothesis of the patterns of ontogenetic development for estimating relative maturity in *Centrosaurus apertus* can be derived from the collection and combination of independent growth characters through a cladistic approach. Substantial non-ontogenetic variability was present in the sample, but a growth signal was still recovered once isolated specimens were removed. The resulting growth series compares favorably to previous hypotheses regarding the growth of *C. apertus* (Sampson, Ryan & Tanke, 1997; Ryan *et al.*, 2001).

(2) Two different coding methods were compared using the same data set. On average, multistate coding generated a more parsimonious (lower CI) and better-resolved tree. A reduced data set of articulated material yielded a single most parsimonious tree using multistate coding. The same reduced data set using binary coding obtained 13 trees that were each 20 steps longer than that recovered by the multistate analysis.

(3) As *C. apertus* developed the nasal horn transitioned from a recurved orientation in the least mature individuals, to a straight orientation in more mature individuals, and finally to a procurved orientation in the most mature individuals. This dramatic change in horn orientation is similar to the ontogenetic transition seen in the supraorbital horns of *Triceratops* (Horner & Goodwin, 2006).

(4) The supraorbital region shows more variability than other areas of the skull. Supraorbital horns were resorbed in the most mature individuals, leaving the area pitted. This pitting is variably developed throughout the ontogram, implying that factors other than ontogeny may contribute to the morphological diversity of this region.

(5) In the most mature specimens, ornamentations may be resorbed so that they are shorter, more rugose in texture, and withered in appearance, in contrast to less mature individuals. This phenomenon is also found in other ceratopsids, pachycephalosaurs, and tyrannosaurids, implying that ontogenetic display structure reduction is a primitive feature for dinosaurs.

(6) Growth characters based upon bone textural changes and bone fusion events are the best proxies for relative development in the skulls of *Centrosaurus apertus*. Ornamentation characters are less congruent with maturity, as these characters vary between individuals, and go through dramatic shifts in relative development, changes in orientation, and resorption throughout life. Size, as determined by frill length, can also justifiably be used to determine general ontogenetic status. The use of size and bone textural changes are the optimum factors for determining the relative maturity in individuals that have yet to grow to adult size. In the largest individuals, these factors differ insufficiently to determine relative maturity. For these individuals, the order of epiparietal and episquamosal fusion (caudal to rostral) along with the orientation of the nasal horn provides a better indication of ontogenetic status.

## Institutional abbreviations

AMNH FARB, American Museum of Natural History, Fossil Amphibians, Reptiles, and Birds, New York, New York; CMN, Canadian Museum of Nature, Ottawa, Ontario; ROM, Royal Ontario Museum, Toronto, Ontario; TMP, Royal Tyrrell Museum of Palaeontology, Drumheller, Alberta; UALVP, The University of Alberta Laboratory of Vertebrate Paleontology, Edmonton, Alberta; YPM, Yale Peabody Museum, New Haven, Connecticut.

## Supplemental Information

10.7717/peerj.252/supp-1 Supplemental Information 1 Reduced matrix based on multistate characters

10.7717/peerj.252/supp-2 Supplemental Information 2 Complete matrix based on multistate characters

10.7717/peerj.252/supp-3 Supplemental Information 3 Reduced matrix based on binary characters

10.7717/peerj.252/supp-4 Supplemental Information 4 Complete matrix based on binary characters

10.7717/peerj.252/supp-5 Supplemental Information 5 Character lists coded in binary and multistate
