# Supplementary material for: Craniofacial ontogeny in Centrosaurus apertus
Source: PeerJ. 2014 Feb 13;2:e252. doi: 10.7717/peerj.252 (PMC3933270; doi:10.7717/peerj.252)
Supplement: Supplemental Information 5 [file peerj-02-252-s005.docx]

Multistate Character list-

The list of hypothetical growth characters. All sutures refer to external only.

Rostral:

1. Rostral, shape: triangular (0), large ventral process or forked (1).
2. Rostral, premaxilla suture: open (0), partially obscured (fused; 1), fully obscured (fully fused; 2).
3. Rostral, texture: smooth (0), rugose and covered in foramina (1).

Premaxilla:

1. Premaxilla, septum, foramen/sulci, presence: absent (0), present (1).
2. Premaxilla, rostrodorsal margin, convexity, presence: absent (0), present (1).
3. Premaxilla, rostrodorsal margin, well-defined sulci, presence: absent (0), present (1).
4. Premaxilla, rostrodorsal margin, texture: smooth (0), rugose (1).
5. Premaxilla, caudal surface of external naris, triangular process, size: absent (0), short (1), Long (2) (Sternberg, 1940).
6. Premaxilla, texture: striated (0), mottled (1), adult (2).
7. Premaxilla, ventral ridge to septum: shallow (0), steep (1).
8. Premaxilla, contact with lacrimal: absent (0), present (1).
9. Premaxilla, suture with opposing premaxilla (rostrodorsally): open (0), partially obscured (fused; 1), fully obscured (fully fused; 2).

Nasal:

1. Nasal, horn orientation: recurved (0), straight (1), or procurved (2).
2. Nasal, horn, position of horn center: caudal to external naris (0), dorsal or rostral to inter-narial process (1) (Dodson et al. 2004).
3. Nasal, horn, length: length is less than twice as long as base is wide (0), length is greater than or equal to twice as long as base is wide (1).
4. Nasal, horn, caudal sulci, presence: absent (0), present, but shallow or not extensive (1), present and extends from the tip to base of the horn (2).
5. Nasal, horn, lateral compression: blade-like (0), expanded and rounded in cross section (1) (Ryan et al. 2001).
6. Nasals, fusion, nasal sutures rostral to nasal horn: unfused (0), partially obscured (fused; 1), fully obscured (fully fused; 2) (Ryan et al. 2001).
7. Nasals, fusion, nasal sutures caudal to nasal horn: unfused (0), partially obscured (fused; 1), fully obscured (fully fused; 2) (Ryan et al. 2001).
8. Nasal, horn, fusion of horncore, extent: unfused (0), only the tip (less than ½ of horn) fused (1), at least ½ of horn fused (2), complete fusion of horn (3).
9. Nasal, horn, constriction at base: unconstricted (0), constricted (1) (Ryan et al. 2001).
10. Nasal, texture: striated (0), not striated (1).
11. Nasal, thickening on the rostrodorsal margin (convexity), presence: absent (0), present (1).

Maxilla:

1. Maxilla, antorbital fenestra, presence: present (0), present but not visible laterally (1), absent (2).
2. Maxilla, accessory fenestra, ventral or rostral to the anorbital fenestra: absent (0), present (1).
3. Maxilla, texture: striated (0), not striated (1).

Jugal:

1. Jugal, distal margin, thickening, horn, presence: absent (0), present (1).
2. Jugal, maxilla border, large convexity, presence: absent (0), present (1).
3. Jugal, suture with epijugal: open (0), partially obscured (fused; 1), fully obscured (fully fused; 2).
4. Jugal, texture: striated (0), mottled (1), adult (2).
5. Jugal, suture with lacrimal: open (0), partially obscured (fused; 1), fully obscured (fully fused; 2).

Supraorbital unit:

1. Supraorbital unit, horns, pitting, extent: absent (0), shallow (<2mm) (1), deep (>2mm) (2).
2. Supraorbital unit, orbit, rostral margin or rim, rugose (must have rugose texture) thickening (antorbital buttress), presence: absent (0), present (1).
3. Supraorbital unit, medial transverse channels, presence: absent (0), present (1) (Ryan et al, 2001).
4. Supraorbital unit, longitudinal grooves: absent (0), present (1) (Ryan et al, 2001).
5. Supraorbital unit, horn, lateral surface texture: smooth (0), rugose (1).
6. Supraorbital, horn, shape: short (0), pyramidal (1), inflated (2), remodeled (3).
7. Supraorbital, horn, direction of apex: caudodorsal (1), dorsal (0) (Ryan et al. 2001).
8. Supraorbital, lacrimal suture with palpebral: unfused (0), partially obscured (fused; 1), fully obscured (fully fused; 2) (Ryan et al. 2001).
9. Supraorbital, prefrontal suture fusion to supraorbital unit: unfused (0), partially obscured (fused; 1), fully obscured (fully fused; 2) (Ryan et al. 2001).
10. Supraorbital, palpebral suture fusion to supraorbital unit: unfused (0), partially obscured (fused; 1), fully obscured (fully fused; 2) (Ryan et al. 2001).
11. Supraorbital, caudal suture with jugal: open (0), partially obscured (fused; 1), fully obscured (fully fused; 2).

Frontal

1. Frontal, fontanelle, depth: shallow (0), deep (1) (Sampson et al. 1997)
2. Frontal, fontanelle, closure: open anteriorly past orbits (0), closed anteriorly (1) (Sampson et al. 1997).
3. Frontal, suture with supraorbital unit: unfused (0), partially obscured (fused; 1), fully obscured (fully fused; 2) (Ryan et al. 2001).
4. Frontals, midline suture: unfused (0), partially obscured (fused; 1), fully obscured (fully fused; 2).

Squamosal:

1. Squamosal, shape: flat (0), concave posterolaterally and convex anteromedially (1). (Sampson et al. 1997).
2. Squamosal, episquamosal one, suture: open (0), partially obscured (fused; 1), or fully obscured (fully fused; 2).
3. Squamosal, episquamosal two, suture: open (0), partially obscured (fused; 1), or fully obscured (fully fused; 2).
4. Squamosal, episquamosal three, suture: open (0), partially obscured (fused; 1), or fully obscured (fully fused; 2).
5. Squamosal, episquamosal four, suture: open (0), partially obscured (fused; 1), or fully obscured (fully fused; 2).
6. Squamosal, undulations, orientation: flat or caudally directed (0), laterally curved (1).
7. Squamosal, undulations, area between, shape: flat (0), concave (1).
8. Squamosal, foramen on lateral side, presence: absent (0), partially opened (1), open (2).
9. Squamosal, second foramen on the lateral side, presence: absent (0), partially open (1), open (2).
10. Squamosal, texture: striated (0), mottled (1), adult or rugose (2).
11. Squamosal, dorsal-most undulation, bifurcation: absent (0), present (1).

Parietal:

1. Parietal, fenestra, size: <49% total sagittal length (0), >50% sagittal length (1) (Sampson et al. 1997).
2. Parietal, process 3, horn presence (taller than base is long): absent (0), present (1) (Sampson et al. 1997).
3. Parietal, process 2, horn, presence: absent (0), present (1).
4. Parietal, process 2, length at least twice as long as base is wide: short (0), long (1) (Ryan et al. 2001).
5. Parietal, process 2, orientation: laterally or caudally (0), medially (1), rostrally (2).
6. Parietal, process 2, caudal sulci: absent (0), shallow (1), deep (1 cm) and extensive (2).
7. Parietal, process 2, texture: smooth (0), rugose (1) (Sampson et al. 1997).
8. Parietal, process 1, number of horns present: 0 (0), 1 (1), 2 (2) (Ryan et al. 2001).
9. Parietal, process 1, length: wider than long (0), longer than wide, but does not extend past midway over the parietal fenestra (1), longer than wide and extends past the midway point over the parietal fenestra (2).
10. Parietal, process 1, deep sulci system (longitudinal grooves), presence (at least 1 cm deep): absent (0), present (1) (Sampson et al. 1997).
11. Parietal, horizontal ramus, ridge between process 1, presence: absent (0), short (less than 1 cm) (1), tall (equal to or greater than 1 cm) (2).
12. Parietal process 1, orientation: caudally/dorsally (0), rostrally (1), ventrally (2).
13. Parietal process 1, texture: undeveloped or smooth (0), rugose (1) withered (2) (Sampson et al. 1997; Ryan et al. 2001).
14. Parietal, cuplike depression between right P1 and left P1: absent or flat (0), slightly concave (1) concave (2) (Ryan et al. 2001).
15. Parietal, posterior margin, thickness between P2 and P3: thin (less than or equal to 10 mm thick) (0), thick (more than 10 mm thick) (1).
16. Parietal, small osseous processes projecting into the fenestra, size: absent (0), short (1mm – 9mm) (1), Long (> 1cm)(2).
17. Parietal, epiparietal 4, suture: open (0), partially obscured (fused; 1), fully obscured (fully fused; 2).
18. Parietal, epiparietal 5, suture: open (0), partially obscured (fused; 1), fully obscured (fully fused; 2).
19. Parietal, epiparietal 6, suture: open (0), partially obscured (fused; 1), fully obscured (fully fused; 2).
20. Parietal, epiparietal 7, suture: open (0), partially obscured (fused; 1), fully obscured (fully fused; 2).
21. Parietal, middle parietal bar, bumps, presence: absent (0), 1-2 (1), 3-6 (2) (Ryan et al. 2001).
22. Parietal, sagittal length/width: more than 1.7 (0), 1.69-1.35 (1) 1.34 to 1.0 (2), .99 or less (3) (Sampson et al. 1997).
23. Parietal, texture: striated (0), mottled (1), adult/rugose (2).

Binary character list-

The list of hypothetical growth characters. All sutures refer to external only.

Rostral:

1. Rostral, shape: triangular (0), large ventral process or forked (1).
2. Rostral, premaxilla suture: open (0), partially obscured (fused) or fully obscured (fully fused; 1).
3. Rostral, premaxilla suture: open or partially obscured (fused; 0), fully obscured (fully fused; 1).
4. Rostral, texture: smooth (0), rugose and covered in foramina (1).

Premaxilla:

1. Premaxilla, septum, foramen/sulci, presence: absent (0), present (1).
2. Premaxilla, rostrodorsal margin, convexity, presence: absent (0), present (1).
3. Premaxilla, rostrodorsal margin, well-defined sulci, presence: absent (0), present (1).
4. Premaxilla, rostrodorsal margin, texture: smooth (0), rugose (1).
5. Premaxilla, caudal surface of external naris, triangular process, size: absent (0), short or long (1) (Sternberg, 1940).
6. Premaxilla, caudal surface of external naris, triangular process, size: absent or short (0), long (1) (Sternberg, 1940).
7. Premaxilla, texture: striated (0), mottled or adult (1).
8. Premaxilla, texture: striated or mottled (0), adult (1).
9. Premaxilla, ventral ridge to septum: shallow (0), steep (1).
10. Premaxilla, contact with lacrimal: absent (0), present (1).
11. Premaxilla, suture with opposing premaxilla (rostrodorsally): open (0), partially obscured (fused) or fully obscured (fully fused; 1).
12. Premaxilla, suture with opposing premaxilla (rostrodorsally): open or partially obscured (fused; 0), fully obscured (fully fused; 1).

Nasal:

1. Nasal, horn orientation: recurved or straight (0), procurved (1).
2. Nasal, horn orientation: recurved (0), straight or procurved (1).
3. Nasal, horn, position of horn center: caudal to external naris (0), dorsal or rostral to inter-narial process (1) (Dodson et al., 2004)
4. Nasal, horn, length: length is less than twice as long as base is wide (0), length is greater than or equal to twice as long as base is wide (1).
5. Nasal, horn, caudal sulci, presence: absent or present, but shallow or not extensive (0), present and extends from the tip to base of the horn (1).
6. Nasal, horn, caudal sulci, presence: absent (0), present, but shallow or not extensive or present and extends from the tip to base of the horn (1).
7. Nasal, horn, lateral compression: blade-like (0), expanded and rounded in cross section (1). Ryan et al. (2001).
8. Nasals, fusion, nasal sutures rostral to nasal horn: unfused or partially obscured (fused; 0), fully obscured (fully fused; 1) (Ryan et al. 2001)
9. Nasals, fusion, nasal sutures rostral to nasal horn: unfused (0), partially obscured (fused) or fully obscured (fully fused; 1) (Ryan et al. 2001)
10. Nasals, fusion, nasal sutures caudal to nasal horn: unfused (0), partially obscured (fused) or fully obscured (fully fused; 1) (Ryan et al. 2001).
11. Nasals, fusion, nasal sutures caudal to nasal horn: unfused or partially obscured (fued; 0), fully obscured (fully fused; 1) (Ryan et al. 2001).
12. Nasal, horn, fusion of horncore, extent: unfused (0), only the tip (less than ½ of horn) fused or at least ½ of horn fused or complete fusion of horn (1).
13. Nasal, horn, fusion of horncore, extent: unfused or only the tip (less than ½ of horn) fused (0), at least ½ of horn fused or complete fusion of horn (1).
14. Nasal, horn, fusion of horncore, extent: unfused or only the tip (less than ½ of horn) fused or at least ½ of horn fused (0), complete fusion of horn (1).
15. Nasal, horn, constriction at base: unconstricted (0), constricted (1) (Ryan et al. 2001)
16. Nasal, texture: striated (0), not striated (1).
17. Nasal, thickening on the rostrodorsal margin (convexity), presence: absent (0), present (1).

Maxilla:

1. Maxilla, antorbital fenestra, presence: present (0), present but not visible laterally or absent (1).
2. Maxilla, antorbital fenestra, presence: present or present but not visible laterally (0), absent (1).
3. Maxilla, accessory fenestra, ventral or rostral to the anorbital fenestra: absent (0), present (1).
4. Maxilla, texture: striated (0), not striated (1).

Jugal:

1. Jugal, distal margin, thickening, horn, presence: absent (0), present (1).
2. Jugal, maxilla border, large convexity, presence: absent (0), present (1).
3. Jugal, suture with epijugal: open or partially obscured (fused; 0), fully obscured (fully fused; 1).
4. Jugal, suture with epijugal: open (0), partially obscured (fused) or fully obscured (fully fused; 1).
5. Jugal, texture: striated (0), mottled or adult (1).
6. Jugal, texture: striated or mottled (0), adult (1).
7. Jugal, suture with lacrimal: open (0), partially obscured (fused) or fully obscured (fully fused; 1).
8. Jugal, suture with lacrimal: open or partially obscured (fused; 0), fully obscured (fully fused; 1).

Supraorbital unit:

1. Supraorbital unit, horns, pitting, extent: absent (0), shallow (<2mm) or deep (>2mm) (1).
2. Supraorbital unit, horns, pitting, extent: absent or shallow (<2mm) (0), deep (>2mm) (1).
3. Supraorbital unit, orbit, rostral margin or rim, rugose (must have rugose texture) thickening (antorbital buttress), presence: absent (0), present (1).
4. Supraorbital unit, medial transverse channels, presence: absent (0), present (1) (Ryan et al, 2001).
5. Supraorbital unit, longitudinal grooves: absent (0), present (1) (Ryan et al, 2001).
6. Supraorbital unit, horn, lateral surface texture: smooth (0), rugose (1).
7. Supraorbital, horn, shape: short (0), pyramidal or inflated or remodeled (1).
8. Supraorbital, horn, shape: short or pyramidal (0), inflated or remodeled (1).
9. Supraorbital, horn, shape: short or pyramidal or inflated (0), remodeled (1).
10. Supraorbital, horn, shape: short or remodeled (0), or pyramidal or inflated (1).
11. Supraorbital, horn, shape: short or remodeled or pyramidal (0), inflated (1).
12. Supraorbital, horn, shape: short or remodeled or inflated (0), pyramidal (1).
13. Supraorbital, horn, direction of apex: caudodorsal (1), dorsal (0) (Ryan et al. 2001).
14. Supraorbital, lacrimal suture with palpebral: unfused (0), partially obscured (fused) or fully obscured (fully fused; 1) (Ryan et al. 2001).
15. Supraorbital, lacrimal suture with palpebral: unfused or partially obscured (fused; 0), fully obscured (fully fused; 1) (Ryan et al. 2001).
16. Supraorbital, prefrontal suture fusion to supraorbital unit: unfused (0), partially obscured (fused) or fully obscured (fully fused; 1) (Ryan et al. 2001).
17. Supraorbital, prefrontal suture fusion to supraorbital unit: unfused or partially obscured (fused; 0), fully obscured (fully fused, 1) (Ryan et al. 2001).
18. Supraorbital, palpebral suture fusion to supraorbital unit: unfused (fused; 0), partially obscured or fully obscured (fully fused; 1) (Ryan et al. 2001).
19. Supraorbital, palpebral suture fusion to supraorbital unit: unfused or partially obscured (fused; 0), fully obscured (fully fused; 1) (Ryan et al. 2001).
20. Supraorbital, caudal suture with jugal: open (0), partially obscured (fused) or fully obscured (fully fused; 1).
21. Supraorbital, caudal suture with jugal: open or partially obscured (fused; 0), fully obscured (fully fused; 1).

Frontal

1. Frontal, fontanelle, depth: shallow (0), deep (1) (Sampson et al. 1997)
2. Frontal, fontanelle, closure: open anteriorly past orbits (0), closed anteriorly (1) (Sampson et al. 1997).
3. Frontal, suture with supraorbital unit: open (0), partially obscured (fused) or fully obscured (fully fused; 1) (Ryan et al. 2001).
4. Frontal, suture with supraorbital unit: open or partially obscured (fused; 0), fully obscured (fully fused; 1) (Ryan et al. 2001).
5. Frontals, midline suture: open (0), partially obscured (fused) or fully obscured (fully fused; 1).
6. Frontals, midline suture: open or partially obscured (fused; 0), fully obscured (fully fused; 1).

Squamosal:

1. Squamosal, shape: flat (0), concave posterolaterally and convex anteromedially (1) (Sampson et al. 1997).
2. Squamosal, episquamosal one, suture: open (0), partially obscured (fused) or fully obscured (fully fused; 1).
3. Squamosal, episquamosal one, suture: open or partially obscured (fused; 0), fully obscured (fully fused; 1).
4. Squamosal, episquamosal two, suture: open (0), partially obscured (fused) or fully obscured (fully fused; 1).
5. Squamosal, episquamosal two, suture: open or partially obscured (fused; 0), fully obscured (fully fused; 1).
6. Squamosal, episquamosal three, suture: open (0), partially obscured (fused) or fully obscured (fully fused; 1).
7. Squamosal, episquamosal three, suture: open or partially obscured (fused; 0), fully obscured (fully fused; 1).
8. Squamosal, episquamosal four, suture: open (0), partially obscured (fused) or fully obscured (fully fused; 1).
9. Squamosal, episquamosal four, suture: open or partially obscured (fused; 0), fully obscured (fully fused; 1).
10. Squamosal, undulations, orientation: flat or caudally directed (0), laterally curved (1).
11. Squamosal, undulations, area between, shape: flat (0), concave (1).
12. Squamosal, foramen on lateral side, presence: absent (0), partially opened or open (1).
13. Squamosal, foramen on lateral side, presence: absent or partially opened (0), open (1).
14. Squamosal, second foramen on the lateral side, presence: absent (0), partially open or open (1).
15. Squamosal, second foramen on the lateral side, presence: absent or partially open (0), open (1).
16. Squamosal, texture: striated (0), mottled or adult or rugose (1).
17. Squamosal, texture: striated or mottled (0), adult or rugose (1).
18. Squamosal, dorsal most undulation, bifurcation: absent (0), present (1).

Parietal:

1. Parietal, fenestra, size: <49% total sagittal length (0), >50% sagittal length (1) (Sampson et al. 1997).
2. Parietal, process 3, horn presence (taller than base is long): absent (0), present (1) (Sampson et al. 1997).
3. Parietal, process 2, horn, presence: absent (0), present (1).
4. Parietal, process 2, length at least twice as long as base is wide: short (0), long (1) (Ryan et al. 2001).
5. Parietal, process 2, orientation: laterally or caudally (0), medially or rostrally (1).
6. Parietal, process 2, orientation: laterally or caudally or medially (0), rostrally (1).
7. Parietal, process 2, caudal sulci: absent (0), shallow or deep (1 cm) and extensive (1).
8. Parietal, process 2, caudal sulci: absent or shallow (0), deep (1 cm) and extensive (1).
9. Parietal, process 2, texture: smooth (0), rugose (1) (Sampson et al. 1997).
10. Parietal, process 1, number of horns present: 0 (0), 1 or 2 (1) (Ryan et al. 2001).
11. Parietal, process 1, number of horns present: 0 or 1 (0), 2 (1) (Ryan et al. 2001).
12. Parietal, process 1, length: wider than long (0), longer than wide, but does not extend past midway over the parietal fenestra or longer than wide and extends past the midway point over the parietal fenestra (1).
13. Parietal, process 1, length: wider than long or longer than wide, but does not extend past midway over the parietal fenestra (0), longer than wide and extends past the midway point over the parietal fenestra (1).
14. Parietal, process 1, deep sulci system (longitudinal grooves), presence (at least 1 cm deep): absent (0), present (1). (Sampson et al. 1997).
15. Parietal, horizontal ramus, ridge between process 1, presence: absent (0), short (less than 1 cm) or tall (equal to or greater than 1 cm, 1).
16. Parietal, horizontal ramus, ridge between process 1, presence: absent or short (less than 1 cm, 0), tall (equal to or greater than 1 cm, 1).
17. Parietal process 1, orientation: caudally/dorsally (0), rostrally or ventrally (1).
18. Parietal process 1, orientation: caudally/dorsally or rostrally (0), ventrally (1).
19. Parietal process 1, texture: undeveloped or smooth (0), rugose or withered (1) (Sampson et al. 1997; Ryan et al. 2001).
20. Parietal process 1, texture: undeveloped or smooth or rugose (0) withered (1) (Sampson et al. 1997; Ryan et al. 2001).
21. Parietal, cuplike depression between right P1 and left P1: absent or flat (0), slightly concave or concave (1) (Ryan et al. 2001).
22. Parietal, cuplike depression between right P1 and left P1: absent or flat or slightly concave (0), concave (1) (Ryan et al. 2001).
23. Parietal, posterior margin, thickness between P2 and P3: thin (less than or equal to 10 mm thick) (0), thick (more than 10 mm thick) (1).
24. Parietal, small osseous processes projecting into the fenestra, size: absent (0), short (1mm – 9mm) or Long (> 1cm) (1)
25. Parietal, small osseous processes projecting into the fenestra, size: absent or short (1mm – 9mm) (0), Long (> 1cm) (1)
26. Parietal, epiparietal 4, suture: open (0), partially obscured (fused) or fully obscured (fully fused; 1).
27. Parietal, epiparietal 4, suture: open or partially obscured (fused; 0), fully obscured (fully fused; 1).
28. Parietal, epiparietal 5, suture: open (0), partially obscured (fused) or fully obscured (fully fused; 1).
29. Parietal, epiparietal 5, suture: open or partially obscured (fused; 0), fully obscured (fully fused; 1).
30. Parietal, epiparietal 6, suture: open (0), partially obscured (fused) or fully obscured (fully fused; 1).
31. Parietal, epiparietal 6, suture: open or partially obscured (fused; 0), fully obscured (fully fused; 1).
32. Parietal, epiparietal 7, suture: open (0), partially obscured (fused) or fully obscured (fully fused; 1).
33. Parietal, epiparietal 7, suture: open or partially obscured (fused; 0), fully obscured (fully fused; 1).
34. Parietal, middle parietal bar, bumps, presence: absent (0), 1-6 (1) (Ryan et al. 2001).
35. Parietal, middle parietal bar, bumps, presence: 0-2 (0), 3-6 (1) (Ryan et al. 2001).
36. Parietal, sagittal length/width: more than 1.7 (0), 1.69 or less (1) (Sampson et al. 1997).
37. Parietal, sagittal length/width: more than 1.35 (0) 1.34 or less (1) (Sampson et al. 1997).
38. Parietal, sagittal length/width: more than 1.0 (0), .99 or less (1) (Sampson et al. 1997).
39. Parietal, texture: striated (0), mottled or adult/rugose (1).
40. Parietal, texture: striated or mottled (0), adult/rugose (1).
